# Mitochondrial DNA reveals secondary contact in Japanese harbour seals, the southernmost population in the western Pacific

**DOI:** 10.1371/journal.pone.0191329

**Published:** 2018-01-31

**Authors:** Mariko Mizuno, Takeshi Sasaki, Mari Kobayashi, Takayuki Haneda, Takahito Masubuchi

**Affiliations:** 1 Graduate School of Bioindustry, Tokyo University of Agriculture, Abashiri, Hokkaido, Japan; 2 Incorporated Non Profit Organization Marine Wildlife Center of Japan, Abashiri, Hokkaido, Japan; 3 Graduate School of Human and Animal-Plant Relationships, Tokyo University of Agriculture, Atsugi, Kanagawa, Japan; National Cheng Kung University, TAIWAN

## Abstract

In this study, we used relatively large number of samples (n = 178) and control region of mtDNA (454bp) to clearify the divergence history of Japanese harbour seals (*Phoca vitulina stejnegeri*) and phylogenetic relationship between the seals in Japan and other countries. Our results suggested that Japanese harbour seals possibly consisted of more than two lineages and secondary contact of populations after a long isolation. Furthermore, one of the lineage was made only by Japanese harbour seals (Group P1). The proportion of Group P1 was the highest at the South West and gradually decreased towards the North East of Hokkaido, Japan. On the other hand, the haplotypes do not belonged to Group P1 showed close relationship to the seals in the North Pacific. Based on the fossil record of harbour seal in Japan and the range of sea ice during the Last Glacial Maximum (LGM), Group P1 might have entered Japan before the LGM and became isolated due to the geographical boundary, and gradually extended its range from the South West towards the North East of Hokkaido after the disappearance of the sea ice, while the seals which are not in Group P1 immigrated into Japan from the North Pacific.

## Introduction

**Fig 1:**
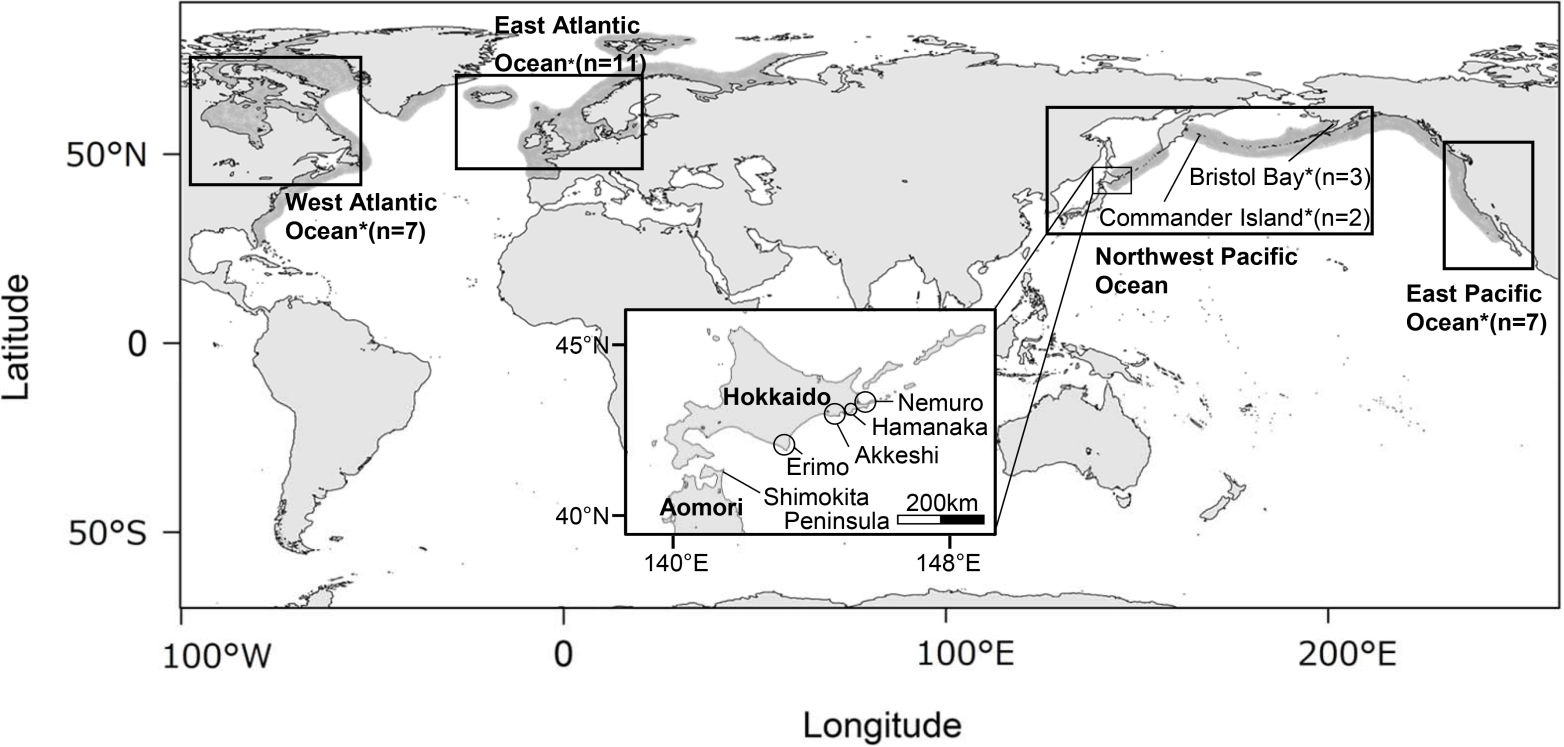
Distribution range of harbour seals (shaded) and sampled localities (squares). Sampling locations of published sequences outside Hokkaido, Japan, used in phylogenetic analysis are indicated with stars (Accession numbers U36342–U36371 [3]). Samples of Japanese harbour seals (*Phoca vitulina stejnegeri*) were taken from four administrative districts (Erimo, Akkeshi, Hamanaka, and Nemuro) in Hokkaido, Japan. Each district contains several haul-out sites where the seals breed.

The harbour seal (*Phoca vitulina*) is an amphibious mammal that distributes across more than 16,000 km of the northern hemisphere (Fig 1). Although their number and division are still a subject of debate, at least four subspecies of harbour seals are known in this range of distribution [1]. Harbour seals are widely distributed along the shore of the Pacific Ocean from Hokkaido, Japan, as the southernmost limit in the western Pacific, to California (*Phoca vitulina richardsi*), the southernmost limit in eastern Pacific [1]. In Japan, harbour seals inhabit only the Pacific side of Hokkaido and are distributed across four administrative districts: Erimo, Akkeshi, Hamanaka, and Nemuro. Akkeshi, Hamanaka, and Nemuro are located next to each other, while Erimo is isolated and 150 km west of Akkeshi, the nearest district (Fig 1)[2].

The common ancestor of the harbour seal diverged 4.5 million years ago from *Pusa* and *Halichoerus* lineages in the area between Greenland and the Barents Sea [4] and entered the Pacific through the Bering Strait. When the northern pathway closed due to the formation of sea ice and continental glaciation 1.7 to 2.2 million years ago, the Atlantic and Pacific harbour seals were separated, eventually resulting in genetic differentiation between the two populations [3].

In the Pacific, harbour seals were said to have colonised from west to east (n = 9) [3], east to west (five additional samples to the same Japanese samples as [3]) [5], or in both directions (same Japanese samples as [3]) [6] which treated Japanese harbour seals as either a basal (ancestral) lineage [3,6] or a non-basal lineage [5] using limited number of samples. On the other hand, the phylogenetic study using only Japanese samples and cytochrome b region of mtDNA suggested there are two lineages (n = 39) [7]. We hypothesed the existence of the different perspectives may be due to handling Japanese harbour seals as a single lineage or not. However, comparisons of all data was not possible because the former studies used control region of mtDNA [3,5,6]. Therefore, the number of lineages and the phylogenetic relationship of Japanese harbour seals with neibouring countries are still unclear.

In this study, our aim was to reach a conclusion concerning the divergence history of Japanese harbour seals and phylogenetic relationship between the seals in Japan and other countries using larger number of samples based on control region of mtDNA. We believe this will help understanding the phylogeny and the historical movement of the Pacific harbour seals as a whole in the future.

## Materials and methods

### Sample area and sample size

Samples were collected from four administrative districts in Hokkaido, Japan: Erimo, Akkeshi, Hamanaka, and Nemuro, each of which has several haulout sites where Japanese harbour seals breed. Three districts are located next to each other (Akkeshi, Hamanaka, and Nemuro), while Erimo is 150 km west of Akkeshi, the nearest district (Fig 1).

A total of 178 samples were collected from the four districts (n = 50 each for Erimo and Nemuro, n = 49 for Akkeshi, and n = 29 for Hamanaka). Muscle samples were taken from dead seals that were incidentally caught in salmon set-nets and drowned (n = 152) or found stranded (n = 7), and skin samples were collected from live animals during the flipper-tagging process for academic research (n = 19). Sample collection from live animals was carried out under the Wildlife Protection and Hunting Management Law; permission numbers obtained from the Ministry of the Environment are: 039 (2009), 001 (2010) and 246 (2012) for eastern Hokkaido, and 291 (2011), 192 (2012), and 0205 (2013) for Erimo. Sampling protocols were approved by the Ethics Committee of Tokyo University of Agriculture. All samples were preserved in 70% ethanol at room temperature until DNA extraction was carried out.

### DNA extraction, PCR, and sequencing

Genomic DNA was extracted from samples using the standard phenol-chloroform method [8]. A total segment of the mtDNA control region was amplified using a polymerase chain reaction (PCR) with primers PvsF (5’-GTACTCATACCCATTGCCAGC-3’) and PvsR (5’-GCGCGGAGGCTTGCATGTAT-3’) designed for this study. PCRs were conducted in a 25 μl reaction volume containing 1.0 μl of DNA template, 2.5 μl 10X buffer, 2.0 μl dNTP (0.2 mM), 0.1 μl taq polymerase (5U/ μl), 1.25 μl (1 mM) of each primer, and 16.9 μl Mili-Q water. MtDNA amplification consisted of an initial denaturation step for 5 min at 94°C, 30 cycles of 94°C for 1 min, 63°C for 1 min, 72°C for 1 min 30 s, and a final extension at 72°C for 5 min. PCR products were checked on agarose gel by electrophoresis and sequenced using a BigDye terminator cycle sequencing kit v3.1 (Applied Biosystems). The same forward primer and an additional reverse primer PvsFR (5’-GTAACGTAACTATGTCCCGC-3’) was used for DNA sequencing, and sequences were read in both directions. Sequence editing and running CLUSTALW for alignment were implemented in MEGA version 6 [9].

A sequence of 454 base pairs (bp) was used for analysis to examine the phylogeny of Japanese harbour seals. Only the data of Stanley et al. [3] (GenBank accession numbers U36342–U36371) was included for the analysis since they have the longest sequence deposited in the GenBank database.

### Data analysis

For the phylogenetic tree, the most appropriate model of substitution was determined using the Baysian Information Criterion (BIC) in MEGA6 [9], and the K2+G+I model was used for the maximum-likelihood (ML) tree. A tree based on the neighbour-joining (NJ) method using same substitution model (K2+G+I) was also created in MEGA6 to validate the phylogenetic tree [9].

To visualise patterns of geographical distribution and haplotype relationships, the median-joining network (MJ Network) was generated using Network 4.6.1.3 [10] with default parameters (epsilon = 0, weight = 10).

The results for the phylogenetic tree and network were combined to examine groupings of Japanese haplotypes. The proportions of haplotypes belonging to the different groups were then compared between the four districts to investigate trends.

Mismatch distribution analysis, which compares the distribution of the observed numbers of pairwise differences among all haplotypes in a sample, was also conducted using Arlequin version 3.5.1.2 [11] to investigate past demographic fluctuations. The goodness of fit between the expected and observed values was tested using the sum of squared deviation (SSD) and Harpending’s raggedness index (Hrag).

## Results

We analysed 454 bp of the mtDNA control region of 178 seals from the four districts of Erimo, Akkeshi, Hamanaka, and Nemuro in Hokkaido, Japan. Overall, 22 polymorphic sites were identified and 16 haplotypes were defined (S1 Table). The haplotypes are deposited to GenBank (accession numbers: LC314221-LC314236)

**Fig 2:**
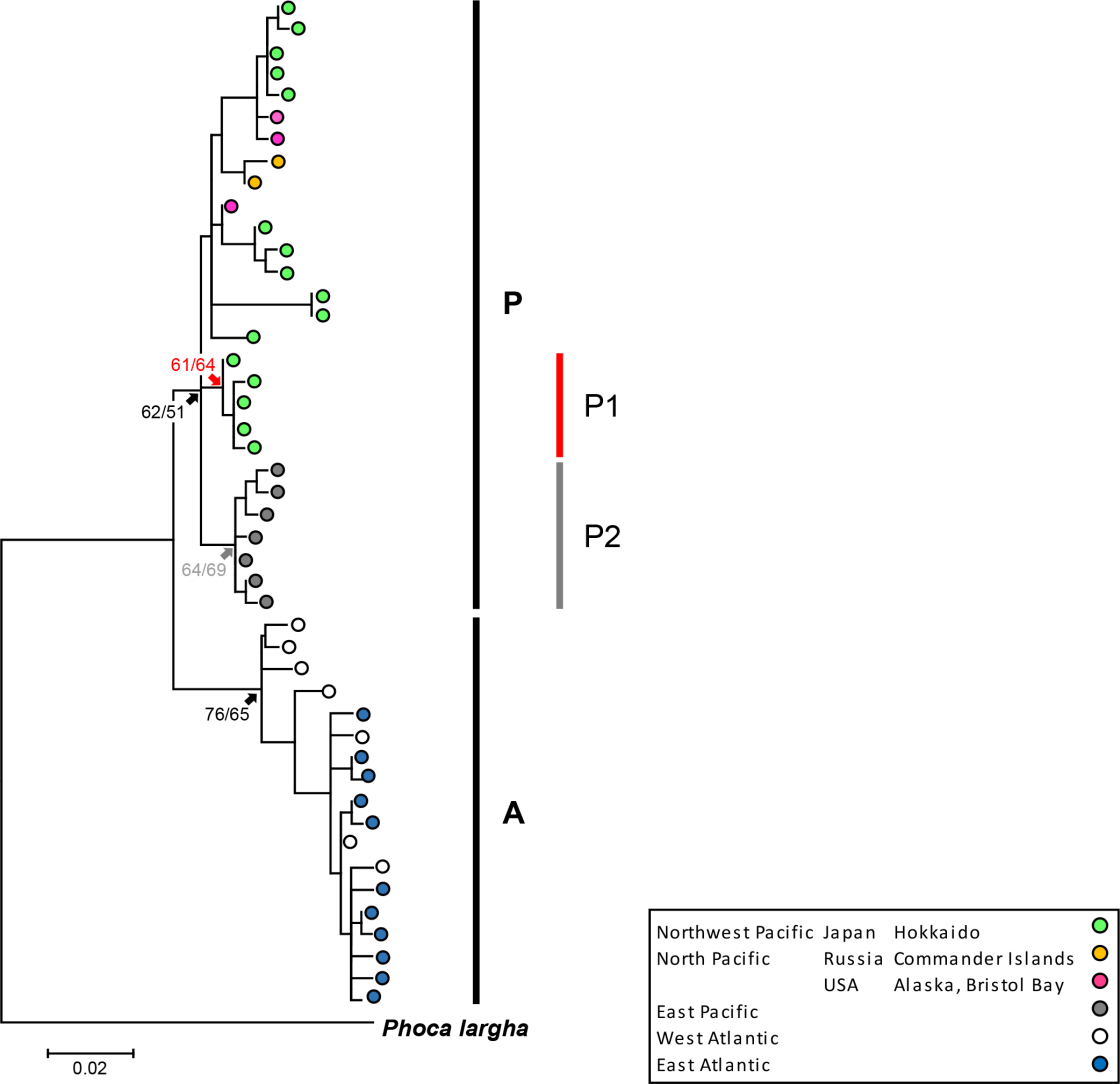
Phylogenetic tree of harbour seals based on the mtDNA control region. The bootstrap values of branches of the maximum-likelihood (left) and neighbour-joining method (right). 454 bp of the control region was used to compare the phylogenetic relationships of seals. Data outside Japan were obtained from GenBank (Accession numbers U36342–U36371 [3]).

Both phylogenetic trees, using the ML and NJ methods, showed the same groupings. A single group was found in the Atlantic (Group A), while Pacific harbour seals (Group P) were divided into a minimum of two groups: the first group only contained haplotypes from Japan (Group P1), and the second group contained haplotypes solely from the eastern Pacific (Group P2) (Fig 2). The Japanese haplotypes other than Group P1 were located in the Group P, along with the haplotypes from Bristol Bay, and the Commander Islands.

**Fig 3:**
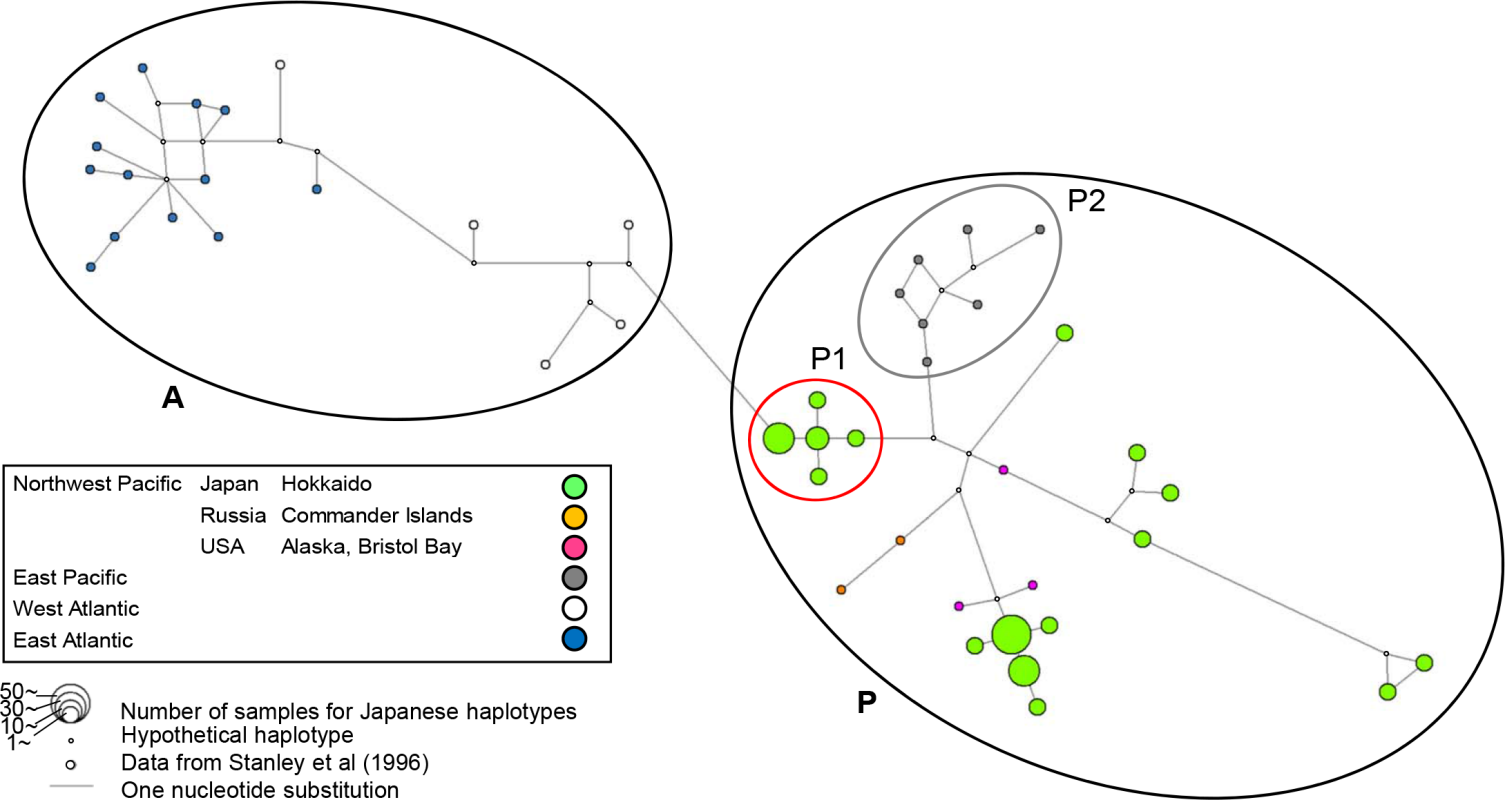
Median-joining network based on the mtDNA control region of harbour seals. The node colours and sizes of circles represent the different sites, area, and sample size. The length of the node is proportional to the number of substitutions. Groupings of the nodes are based on the division of the phylogenetic tree in Fig 2.

Groupings in the median-joining network were conducted based on the phylogenetic tree (Fig 3). In the haplotype network, Group P1 was connected to Atlantic, and contained only Japanese haplotypes. The haplotypes in the eastern Pacific (Group P2) and other haplotypes were then connected to Group P1. The Japanese haplotypes other than Group P1 are located in separate branches, suggesting that they diverged from multiple haplotypes: some were from Bristol Bay and others were from the same hypothetical haplotypes shared with Bristol Bay and the Commander Islands.

**Fig 4:**
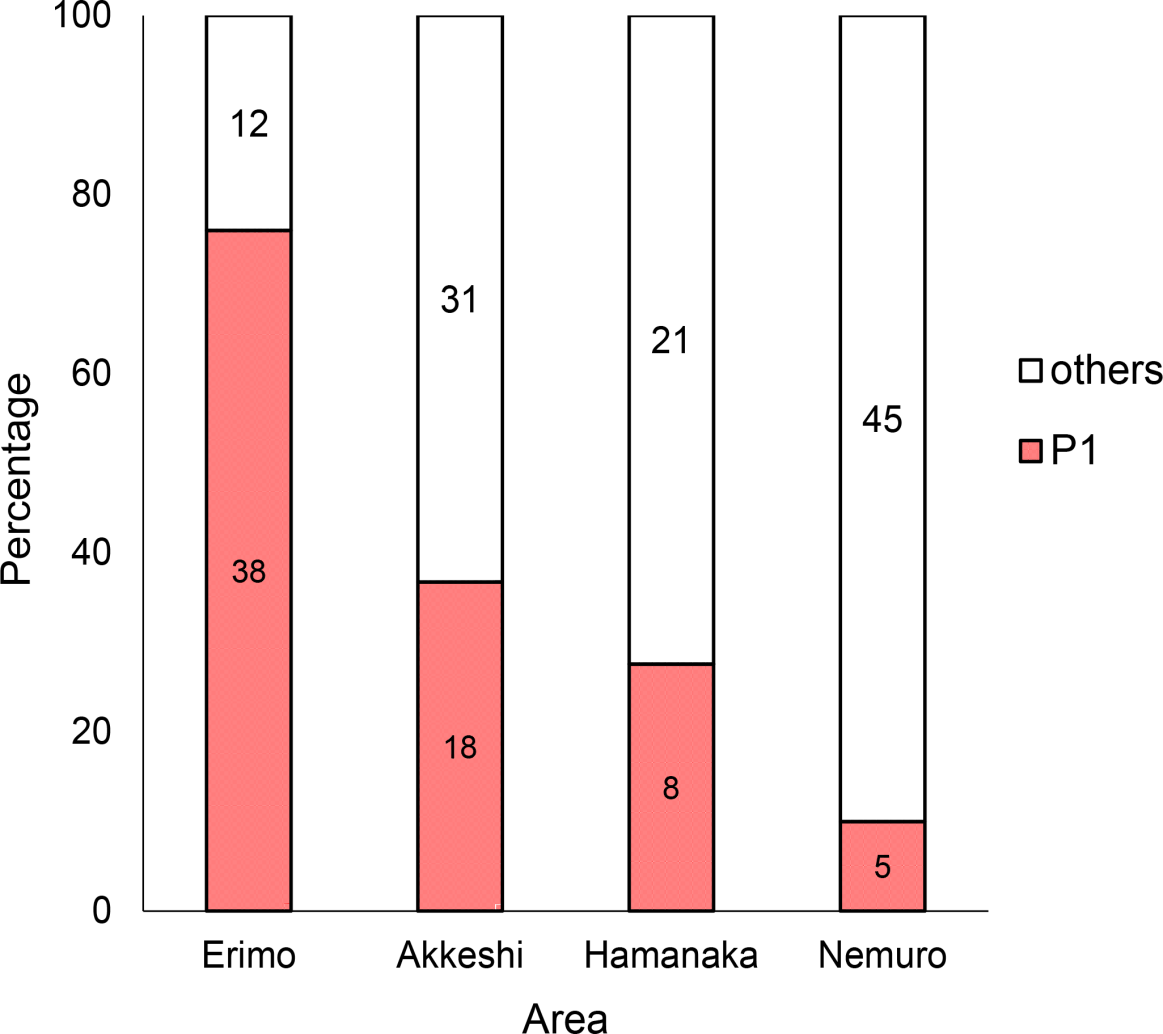
Proportions of haplogroups among the four districts. The haplogroups (Group P, A, P1 and P2) were defined in the phylogenetic tree and the median-joining network. The numbers in the bar indicate the number of samples.

The proportion of Group P1 was high at Erimo, the southernmost distribution in the range of harbour seals in the western Pacific, and decreased toward Nemuro, the easternmost sampling site in this study (Fig 4).

**Fig 5:**
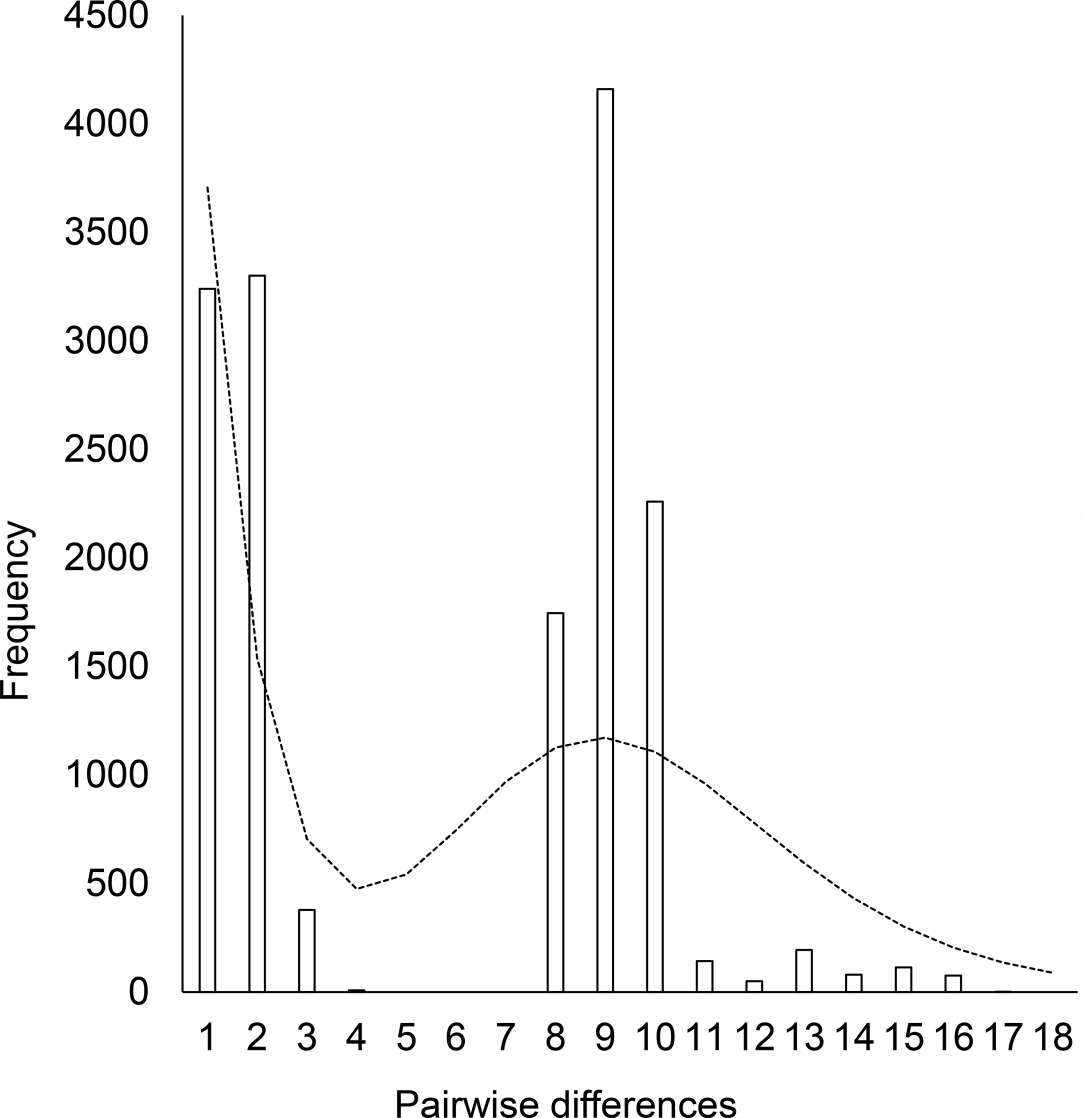
Mismatch distribution of mtDNA haplotypes for Japanese harbour seals. The bar charts indicate the observed number of pairwise differences and the dashed line represents the expected distribution under a spatial expansion model (SSD: p = 0.07; Hrag: p = 0.41).

Mismatch distribution of Japanese harbour seals showed a bimodal profile, indicating secondary contact of populations after a long isolation. SSD and Hrag both supported the overall pairwise differences in the match spatial distribution model (SSD: p = 0.07; Hrag: p = 0.41) (Fig 5) but it did significantly deviated from expectations under a sudden expansion model (SSD: p = 0.02; Hrag: p = 0.02).

## Discussion

Past phylogenetic studies of harbour seals treated Japanese haplotypes as a single lineage. Some concluded that the direction of expansion occurred from west to east and that the seals in Japan represented a basal population in the Pacific [3,6], while another study suggested that population expansion occurred in the opposite direction and that the population in Japan was not basal [5] using control region of mtDNA. On the other hand, other study used only Japanese samples and cytochrome b region of mtDNA suggested existence of two lineages [7]. The number of lineages and the phylogenetic relationship of Japanese harbour seals with neibouring countries were still unclear, because comparison of these studies was not possible.

We used relatively large number of samples (n = 178) and control region of mtDNA (454bp) to clearify the divergence history of Japanese harbour seals and phylogenetic relationship between the seals in Japan and other countries.

Our results indicated there possibly are more than two lineages in Japanese harbour seals based on phylogenetic tree and haplotype network. Also, the mismatch analysis suggested secondary contact of populations after a long isolation; and increase in the population range over time and space after the restriction of original population into a very small area.

Moreover, one of the lineage was made only by Japanese harbour seals (Group P1). The proportion of this lineage was the highest at Erimo, the southernmost distribution range of western Pacific harbour seals and gradually decreased towards the North East of Hokkaido. The Japanese haplotypes which are not in Group P1 (Figs 2 and 3) belonged to different branches, which also had haplotypes from the North Pacific suggested they have close relationship to the Northern Pacific harbour seals.

We further constructed two median joining trees using different data (S1 Fig; data of Fig 3 and [5] (369bp) and S2 Fig; data of Fig 3 and all other data available in GenBank (370bp)) to compare with the haplotype network in result section (Fig 3). All new networks and Fig 3 showed same groupings for Japan (Group P1) and Washington (Group P2) (Fig 3, S1 and S2 Figs), and the other Japanese haplotypes showed close relationship to the seals in North Pacific.

During the Last Glacial Maximum (LGM) which was ended around 0.02 million years ago, the lowering of the sea level and the formation of the Bering land bridge connecting Eurasia and North America caused closure of Bering Strait [12,13]. At this time, the Cordilleran ice sheet covered most of North America, including the eastern Aleutian Islands but not some parts of eastern Alaska and the land bridge over the Bering Strait (Beringia) [12,14]. In addition, seasonal sea ice was extending its range from north to south in the Pacific, to as far as Erimo in Hokkaido, Japan [12,15,16]. The animals lived over the North Pacific during this period are believed to be surviving in small, ice-free regions called refugia, and population subdivision related to refugia across the North Pacific are known in many marine and land animals (e.g. the Steller sea lion [17,18], sea otter [19], rock ptarmigan [20], and reindeer [21], as well as in subspecies of harbour seals in the eastern Pacific [22,23]). The phylogenetic studies of chum salmon [24–27], and Pacific cod [28], which are also distributed widely over the North Pacific, suggested that animals in Hokkaido became isolated during the LGM [24–26,29,30].

Fossils of harbour seals dated as 0.1 million years old were found at the Shimokita Peninsula, Aomori, which is not far from Erimo, currently the southernmost distribution range of harbour seals in the western Pacific (Fig 1) [31,32]. This suggests that harbour seals already inhabited areas around Aomori long before the LGM.

These factors suggest the history of Japanese harbour seals: the haplogroup made up only by Japanese harbour seals (Group P1) might have entered Japan before the LGM and became isolated due to the geographical boundary-sea ice, and gradually extended its range from the South West towards the North East of Hokkaido after the disappearance of the sea ice, while the seals which are not in Group P1 immigrated into Japan from the North Pacific, which are the descendent of the seals in refugia in North Pacific.

## Supporting information

S1 Table Polymorphic sites of the mtDNA control region detected in Japanese harbour seals. (PPTX)

S1 Fig Median joining tree based on the haplotypes of the Pacific harbour seals from Westlake and O’Corry-Crowe (2002), our data and Stanley *et al* (1996). Final 369bp of 255 haplotypes were used after alignment. Colouration for the haplotypes of our data and Stanley et al(1996) are same as Fig 3 for comparison. Haplotypes of Westlake and O’Corry-Crowe (2002) are shown as yellow.
(PDF)

S2 Fig Median joining tree based on all harbour seals data available in GenBank. Final sequences of 356bp 381haplotypes were used after alignment [3,5,6,23,33,34]. Colouration for the haplotypes of our data and Stanley et al(1996) are same as Fig 3 for comparison. Haplotypes of other studies were divided into Atlantic (purple) and Pacific (yellow).
(PDF)
