# Supplementary material for: Mitochondrial DNA reveals secondary contact in Japanese harbour seals, the southernmost population in the western Pacific
Source: PLoS One. 2018 Jan 31;13(1):e0191329. doi: 10.1371/journal.pone.0191329 (PMC5792009; doi:10.1371/journal.pone.0191329)
Supplement: S1 Table — (PPTX) [file pone.0191329.s001.pptx]

## Slide 1
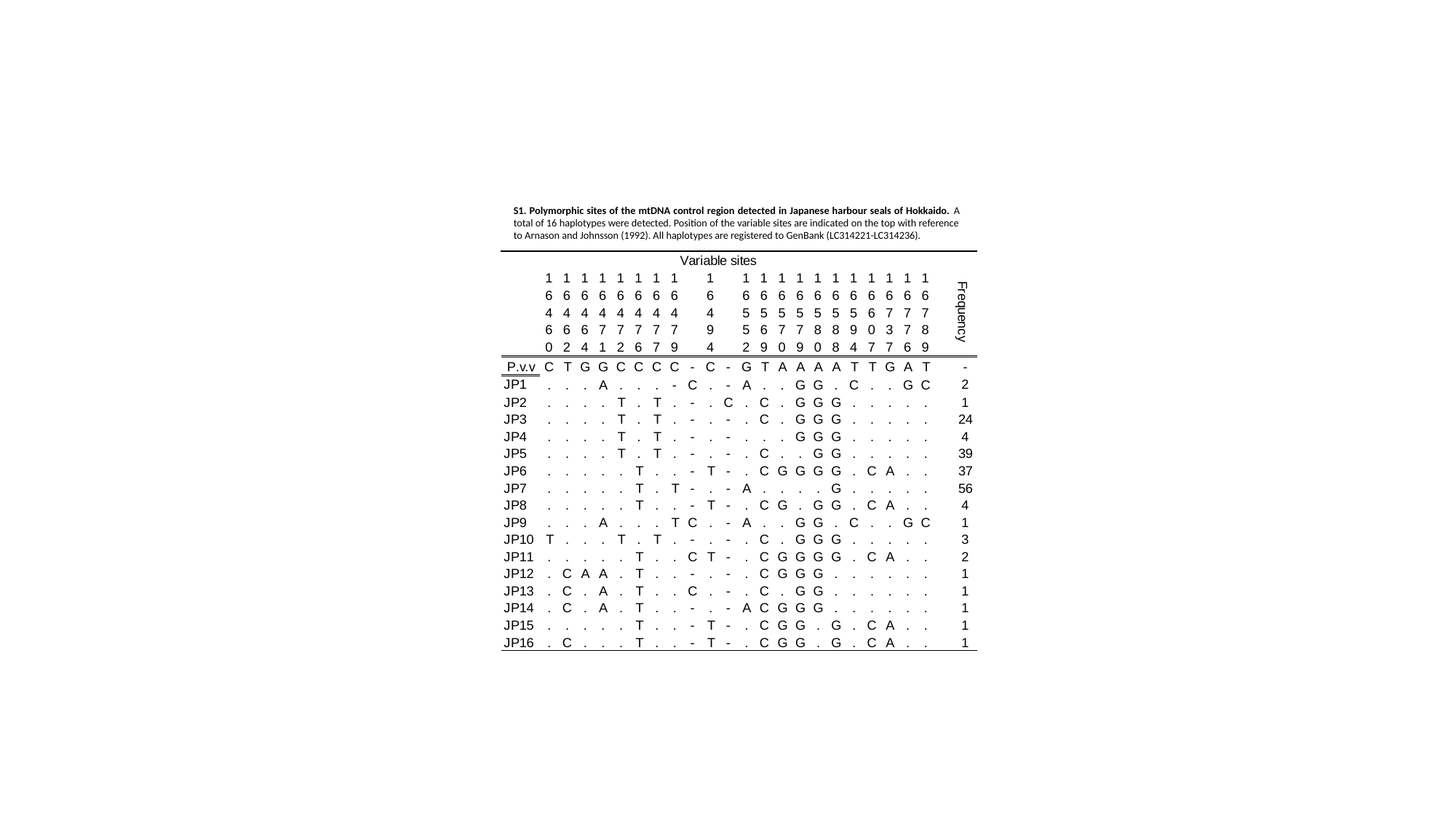

S1. Polymorphic sites of the mtDNA control region detected in Japanese harbour seals of Hokkaido. A total of 16 haplotypes were detected. Position of the variable sites are indicated on the top with reference to Arnason and Johnsson (1992). All haplotypes are registered to GenBank (LC314221-LC314236).
