# Supplementary material for: Mitochondrial DNA reveals secondary contact in Japanese harbour seals, the southernmost population in the western Pacific
Source: PLoS One. 2018 Jan 31;13(1):e0191329. doi: 10.1371/journal.pone.0191329 (PMC5792009; doi:10.1371/journal.pone.0191329)
Supplement: S1 Fig — Final 369bp of 255 haplotypes were used after alignment. Colouration for the haplotypes of our data and Stanley et al(1996) are same as Fig 3 for comparison. Haplotypes of Westlake and O’Corry-Crowe (2002) are shown as yellow. (PDF) [file pone.0191329.s002.pdf]

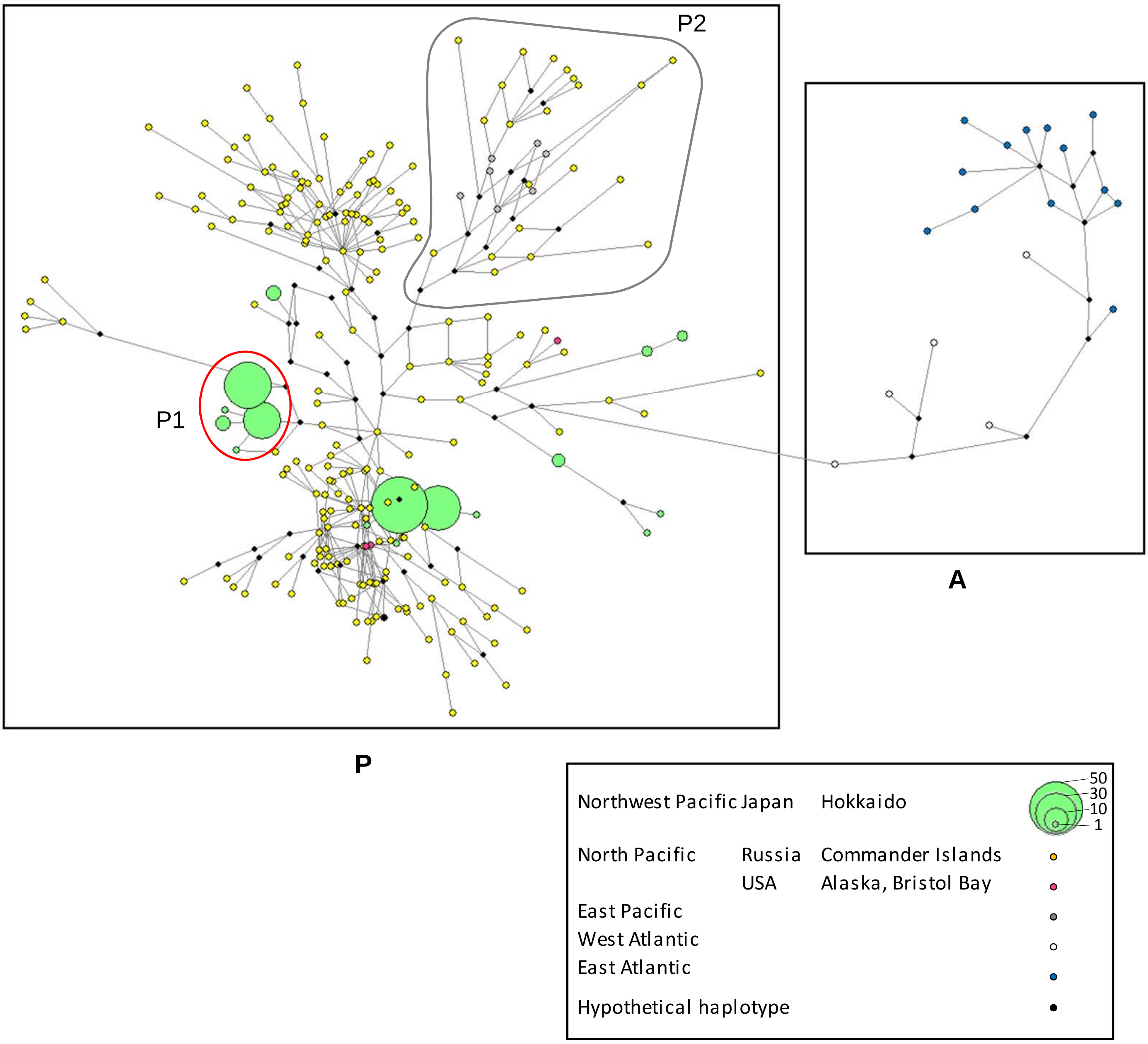

P2
P1
A
P
Northwest Pacific Japan Hokkaido
50
30
10
1
North Pacific Russia Commander Islands
USA Alaska, Bristol Bay
East Pacific
West Atlantic
East Atlantic
Hypothetical haplotype
