# Supplementary figures and images for: Mitochondrial DNA reveals secondary contact in Japanese harbour seals, the southernmost population in the western Pacific
Source: PLoS One. 2018 Jan 31;13(1):e0191329. doi: 10.1371/journal.pone.0191329 (PMC5792009; doi:10.1371/journal.pone.0191329)

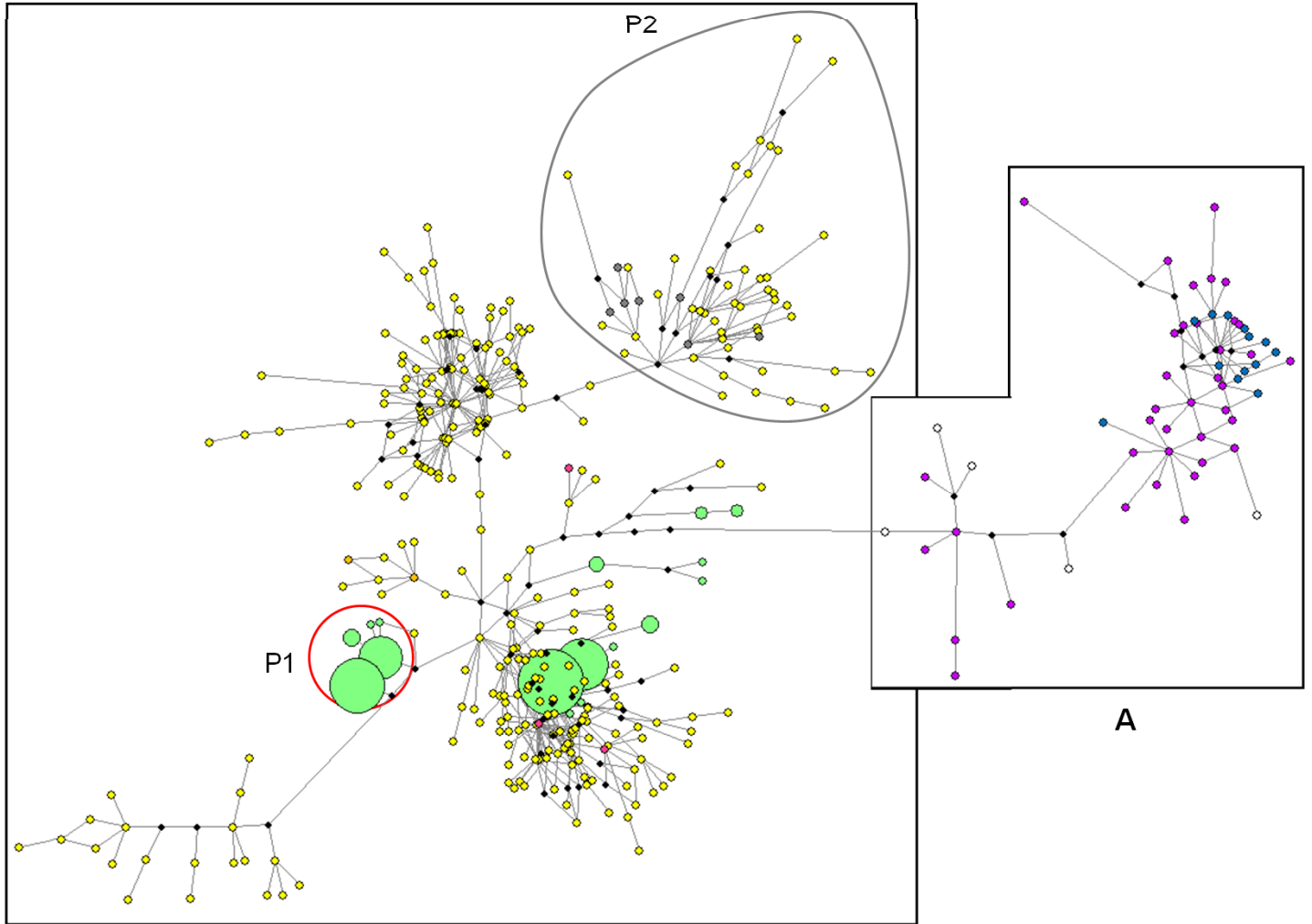

P

A

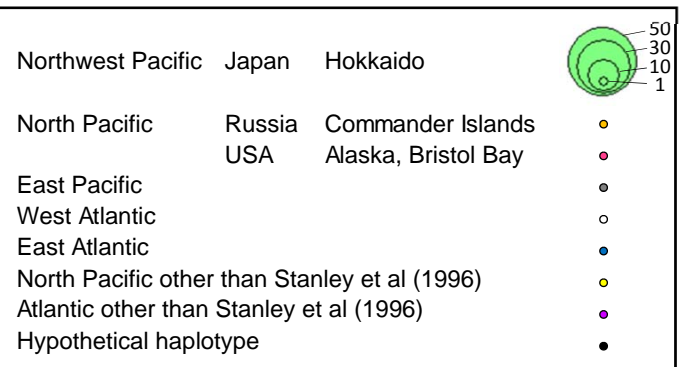

Supplement: S2 Fig — Final sequences of 356bp 381haplotypes were used after alignment [3,5,6,23,33,34]. Colouration for the haplotypes of our data and Stanley et al(1996) are same as Fig 3 for comparison. Haplotypes of other studies were divided into Atlantic (purple) and Pacific (yellow). (PDF) [file pone.0191329.s003.pdf]
